# Loss or major reduction of umami taste sensation in pinnipeds

**DOI:** 10.1007/s00114-012-0939-8

**Published:** 2012-07-10

**Authors:** Jun J. Sato, Mieczyslaw Wolsan

**Affiliations:** 1 Laboratory of Animal Cell Technology, Faculty of Life Science and Technology, Fukuyama University, Higashimura-cho, Aza, Sanzo, 985, Fukuyama, 729-0292 Japan; 2 Museum and Institute of Zoology, Polish Academy of Sciences, Wilcza 64, 00-679 Warszawa, Poland

**Keywords:** Diet, Feeding behavior, Feeding ecology, Marine adaptation, Marine environment

## Abstract

Umami is one of basic tastes that humans and other vertebrates can perceive. This taste is elicited by L-amino acids and thus has a special role of detecting nutritious, protein-rich food. The T1R1 + T1R3 heterodimer acts as the principal umami receptor. The T1R1 protein is encoded by the *Tas1r1* gene. We report multiple inactivating (pseudogenizing) mutations in exon 3 of this gene from four phocid and two otariid species (Pinnipedia). Jiang et al. (Proc Natl Acad Sci U S A 109:4956–4961, 2012) reported two inactivating mutations in exons 2 and 6 of this gene from another otariid species. These findings suggest lost or greatly reduced umami sensory capabilities in these species. The widespread occurrence of a nonfunctional *Tas1r1* pseudogene in this clade of strictly carnivorous mammals is surprising. We hypothesize that factors underlying the pseudogenization of *Tas1r1* in pinnipeds may be driven by the marine environment to which these carnivorans (Carnivora) have adapted and may include: the evolutionary change in diet from tetrapod prey to fish and cephalopods (because cephalopods and living fish contain little or no synergistic inosine 5′-monophosphate that greatly enhances umami taste), the feeding behavior of swallowing food whole without mastication (because the T1R1 + T1R3 receptor is distributed on the tongue and palate), and the saltiness of sea water (because a high concentration of sodium chloride masks umami taste).

## Introduction

The sense of taste is a chemosensory system that identifies nutritionally relevant and harmful substances in food and thus guides organisms to consume or avoid potential food sources. Humans and other vertebrates can detect several taste qualities, including bitter, salty, sour, sweet, and umami. The taste of umami (which means “delicious flavor”) allows the recognition of L-amino acids, the building blocks of proteins, and therefore plays a special role in identifying nutritious food (Chandrashekar et al. 2006; Bachmanov and Beauchamp 2007; Temussi 2009; Yarmolinsky et al. 2009). A conspicuous feature of umami taste is its impressive potentiation by purine nucleotides such as inosine 5′-monophosphate (IMP) and guanosine 5′-monophosphate (GMP; Kuninaka 1960). Several candidate umami receptors have been proposed, including mGluR4 (Chaudhari et al. 2000), T1R1 + T1R3 (Li et al. 2002; Nelson et al. 2002), and mGluR1 (San Gabriel et al. 2005). The T1R1 + T1R3 heterodimer responds to a broad spectrum of L-amino acids (Nelson et al. 2002), shows a strongly potentiated response in the presence of IMP or GMP (Li et al. 2002; Nelson et al. 2002), and is widely regarded as the prototypic umami receptor (Temussi 2009). This receptor is embedded in the membrane of taste receptor cells, which are assembled into taste buds distributed in papillae of the tongue and palate epithelium (Chandrashekar et al. 2006; Yarmolinsky et al. 2009). The T1R1 and T1R3 proteins are encoded by the *Tas1r1* (*TAS1R1*) and *Tas1r3* (*TAS1R3*) genes, respectively (Bachmanov and Beauchamp 2007), the latter gene being also expressed in a T1R2 + T1R3 heterodimer, which functions as a sweet receptor (Nelson et al. 2001; Li et al. 2002).

Pseudogenization is an evolutionary phenomenon in which a gene loses its function due to a disruption in its coding or regulatory sequence (Grus and Zhang 2008). Frameshift insertions, frameshift deletions, and nonsense substitutions introduce stop codons that disrupt the open reading frame of a gene and thereby cause a premature termination of translation of nucleic acids into protein. If such a mutation occurs in *Tas1r1*, this gene will become a pseudogene and will no longer be able to produce a complete functional T1R1 protein. This will result in a dysfunctional T1R1 + T1R3 receptor and can eventually lead to the loss of umami perception. Most vertebrates appear to have a functional (intact) *Tas1r1* (Shi and Zhang 2006; Nei et al. 2008). This gene is known to be inactivated by pseudogenization or absent in the tongueless western clawed frog (Shi and Zhang 2006); some fructivorous, insectivorous, and vampiric bats (Zhao et al. 2012); folivorous giant panda (Li et al. 2010; Zhao et al. 2010); and two marine carnivorous mammals (bottlenose dolphin and California sea lion; Jiang et al. 2012).

Here, we report multiple pseudogenizing mutations in exon 3 of *Tas1r1* from six species of Pinnipedia. Jiang et al. (2012) reported two other pseudogenizing mutations in exons 2 and 6 of this gene from another pinniped species (California sea lion). These findings suggest loss or major reduction of umami sensation in these species. As pinnipeds are strictly carnivorous and entirely depend on a diet rich in proteins, the widespread occurrence of a *Tas1r1* pseudogene in this clade is not expected. We hypothesize that factors underlying the pseudogenization of *Tas1r1* in these secondarily adapted marine carnivorans (Carnivora) may be driven by the marine environment.

## Materials and methods

**Fig. 1:**
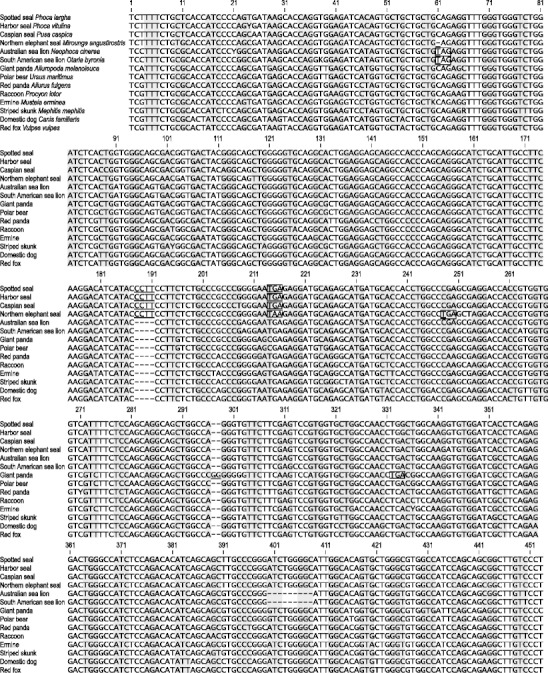
Alignment of a DNA sequence segment from *Tas1r1* exon 3 in 14 carnivorans. Inactivating (pseudogenizing) mutations are *underlined*. Premature stop codons introduced by these mutations are *boxed*. *Gray shading* shows the correct open reading frame. *Dashes* point to alignment gaps. *Numbers* indicate nucleotide positions in this alignment

Partial DNA sequences of *Tas1r1* exon 3 were newly determined for six pinnipeds and six other carnivorans. These sequences have been deposited in the DDBJ/EMBL/GenBank databases with accession numbers AB697513–AB697524. Homologous sequences for two other carnivorans, a polar bear (HM468451) and a domestic dog (HM468447), were obtained from the DDBJ/EMBL/GenBank databases (Fig. 1).

Total genomic DNA was extracted from either tissue or blood using a standard phenol–chloroform method or a DNeasy Blood and Tissue Kit (Qiagen). Amplification was performed through PCR reactions with a KOD-plus-Neo DNA polymerase kit (Toyobo) in an automated thermal cycler (model PC 302, Astec). Each PCR mix contained KOD-plus-NEO buffer, 1.5 mM MgSO_4_, 0.2 mM of each dNTP, 0.3 μM of each of Tas1r1_ex3_Fw1 (5′-GGAGTGAAGCGGTATTACCC-3′) and Tas1r1_ex3_Rv1 (5′-GGCCTCTTCAAACTCCTTCAGGC-3′) primers (both newly designed), 1.0 U of KOD-Plus-Neo DNA polymerase, and 0.1–0.2 μg of template total genomic DNA in a total volume of 50 μl. The PCR thermal cycling parameters included a 2-min denaturation period at 94 °C followed by 35 cycles of denaturation at 98 °C for 10 s, annealing at 50 °C for 30 s, and extension at 68 °C for 30 s; this was followed by a 10-min extension period at 68 °C. Sequencing reaction was carried out with a Big Dye Terminator (v. 3.1) Cycle Sequencing Kit (Applied Biosystems). Sequences were resolved using an ABI3130 automated sequencer (Applied Biosystems).

Multiple sequence alignment was accomplished in MEGA 5 (Tamura et al. 2011). Cladistic analysis was conducted in GARLI 2.0 (Zwickl 2006) using maximum likelihood with the HKY + Γ model of DNA substitution. This optimal model was identified with the Akaike information criterion implemented in Modeltest 3.7 (Posada and Crandall 1998). Insertions were not considered. Deletions and nonsense substitutions were coded as missing data. Both canids (domestic dog and red fox) were used as an outgroup. Tree searching was heuristic. Five runs of the genetic algorithm were performed, each with 50,000 generations of a mutation–selection–reproduction cycle. Starting trees were generated through stepwise addition. Bootstrap percentages were computed from 1,000 pseudoreplicates.

## Results

**Fig. 2:**
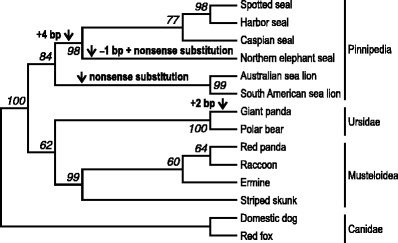
Maximum likelihood phylogeny of 14 carnivorans inferred from sequences tabulated in Fig. 1. *Arrows* indicate branches where pseudogenizing frameshift insertions (+4 bp and +2 bp), frameshift deletion (–1 bp), and nonsense substitutions are hypothesized to have evolved. *Numbers in italics* represent bootstrap percentages for the respective nodes

Examination of a 453-bp aligned segment of *Tas1r1* exon 3 revealed a disrupted open reading frame in all six pinnipeds and the giant panda, and an intact open reading frame in seven other carnivorans. The pseudogenizing mutations observed in the pinnipeds included a 4-bp frameshift insertion (positions 188–191; Fig. 1) in four phocids (spotted seal, harbor seal, Caspian seal, and northern elephant seal), a 1-bp frameshift deletion (position 61) and a nonsense substitution (position 248) in one phocid (northern elephant seal), and a nonsense substitution (position 61) in two otariids (Australian sea lion and South American sea lion). A 2-bp frameshift insertion previously reported from the giant panda (Li et al. 2010; Zhao et al. 2010) was confirmed (positions 297–298). Each of these mutations introduced a premature stop codon (Fig. 1). Phylogenetic analysis demonstrated that the 4-bp insertion first occurred in a common ancestor of the four phocids and was followed by the 1-bp deletion and nonsense substitution in the northern elephant seal lineage, and that the nonsense substitution found in the two otariids arose in their common ancestor (Fig. 2).

## Discussion

The efficient gain of relevant nutrients and avoidance of harmful compounds are essential for the survival and propagation of organisms, and therefore, dietary, feeding behavior, and taste perception changes in organismal evolution are likely to be responded to and reflected in the evolution of genes encoding taste receptor proteins. Pinnipeds mostly feed on fish and cephalopods, and typically consume prey that is small enough to be swallowed whole (Berta et al. 2006). IMP (which greatly enhances the taste of umami) is abundant in muscles and other tissues of tetrapods, but is scarce or absent in cephalopods and living fish (Arai and Saito 1961; Arai 1966; Yamaguchi and Ninomiya 2000; Kurihara 2009). This synergistic compound gradually accumulates postmortem in fish tissues as a result of degradation of adenosine 5′-triphosphate, so that umami taste can eventually be perceived some time after the fish’s death (Arai and Saito 1961; Arai 1966; Kurihara 2009). Furthermore, a high concentration of sodium chloride masks umami taste (Ikeda 1909; Komata 1990). We therefore hypothesize that factors underlying the inactivation of *Tas1r1* in pinnipeds may be driven by the marine environment to which these carnivorans have adapted and may include: the evolutionary change in diet from tetrapod prey to fish and cephalopods, the feeding behavior of swallowing food whole without mastication (also suggested by Jiang et al. 2012), and the saltiness of sea water.

The ability of pinnipeds to sense umami has not been examined. Anatomical studies, however, have shown that gustatory papillae on the tongue of pinnipeds are reduced in number and simplified as compared with other carnivorans (Sonntag 1923; Kubota 1968; Yoshimura et al. 2002). These observations concur with our finding of the widespread pseudogenization of *Tas1r1* in pinnipeds, which suggests lost or at least greatly reduced umami sensory capabilities.
